# The Queensland Virtual Integrated Practice (VIP) partnership program pilot study: an Australian-first model of care to support rural general practice

**DOI:** 10.1186/s12913-023-10189-0

**Published:** 2023-10-31

**Authors:** Breanna Lepre, Jennifer Job, Zena Martin, Natalie Kerrigan, Claire Jackson

**Affiliations:** 1 Centre for Health System Reform and Integration, University of Queensland-Mater Research Institute (UQ/MRI), Royal Brisbane Hospital, Level 8, Health Sciences Building, Herston, QLD 4029 Australia; 2 School of Human Movement and Nutrition Sciences, University of Queensland, St Lucia, QLD 4067 Australia; 3 Health Workforce Queensland, Level 4, 348 Edward Street, Brisbane, QLD 4000 Australia; 4 Western Queensland Primary Health Network, 11 Barkly Hwy, Mount Isa 4825, Miles End QLD, Australia; 5 General Practice Clinical Unit, School of Medicine, University of Queensland, Royal Brisbane Hospital, Level 8, Health Sciences Building, Herston, QLD 4029 Australia

**Keywords:** Workforce crisis, Rural GP workforce, Virtual Integrated Practice, Telehealth, Rural and remote health, Health services research

## Abstract

**Background:**

There is a critical lack of medical workforce internationally, and this is particularly notable in rural and remote Australia where strategies to address workforce shortages are urgently required. This pilot study aimed to implement and evaluate a Virtual Integrated Practice (VIP) Program in the Australian rural primary care setting.

**Methods:**

The VIP model was developed using co-creation methodology and involves an urban GP joining a rural general practice team to provide ongoing care to patients remotely via secure telehealth. The pilot study was conducted in two western Queensland general practices, commencing in October 2021 with one rural practice and extending to an additional rural practice from November 2022. Evaluation included a retrospective review of service, billing and cost data, and an online survey for patients. Ethical approval was obtained from the University of Queensland Human Research Ethics Committee (Project number: 2021/HE002434).

**Results:**

There were 1468 services provided through to December 2022, including general consults (n = 1197), therapeutic procedures (n = 68), mental health treatment plans (n = 68) and chronic disease management plans (n = 59). Patients were predominantly female (73.1%) and did not have their appointment at the practice (57.8%). Among 1282 occasions of service, less than 20% of consultations (n = 224) required support from staff (e.g., a nurse), and more than half were repeat patient encounters (53.0%). Survey respondents (n = 45) indicated that they were satisfied (9.3%) or highly satisfied (90.7%) with the care provided, and importantly, 95.5% of respondents reported that the service improved their access to the GP. More than 20% of respondents indicated that they would attend the Emergency Department if virtual care was not available.

**Conclusions:**

Data from this pilot study has informed translation to an additional 20 vulnerable rural general practices in three further rural regions in Queensland in 2023 and evaluation is ongoing. This pilot study demonstrates the feasibility and acceptability of an innovative, digitally supported community-focussed, healthcare initiative to arrest the decline in rural general practice workforce, improve patient care access and support rural practice viability.

**Supplementary Information:**

The online version contains supplementary material available at 10.1186/s12913-023-10189-0.

## Background

Primary health care (PHC) is at the frontline of Australia’s health care system and remains the cornerstone of efforts to improve health across diverse population groups. Currently however, there is a critical lack of GP workforce internationally, underpinned by widespread dissatisfaction and burnout due to factors such as a lack of resources, inadequate renumeration and an intolerable workload [1, 2]. These shortages are reflected in health modelling, with a projected shortfall of 11,392 general practitioners (GPs), or 1 in 3 of the GP workforce, expected over the next 10 years [3]. Conversely, there is a rising demand for care, underpinned by a rapidly ageing population, and the exigent burden of chronic disease. A 38% increase in demand for GP services in Australia is expected by 2032 [3].

Furthermore, the existing geographic spread of medical workforce does not reflect the distribution of the population, nor the level of care required. Despite a decade of government investment in recruitment and retention strategies, areas of regional and rural Australia are experiencing a critical shortage of GPs. As a result, general practices in rural and remote communities are increasingly non-viable, with many closing its doors [4]. This leaves patients with no other option than to seek care from already overstretched emergency departments, or travel to city or major regional centres for care. A 2020 report found that almost 10% of people living in inner regional, outer regional, remote, or very remote Australia, had no access to any primary healthcare services within a 60-minute drive time, and this inequity is compounded by the lack of medical workforce [5]. As Australians living in rural and remote areas have higher rates of disease, potentially preventable hospitalisations, and deaths, they are particularly at risk [6]. The internationally-supported Quadruple Aim underscores the need to improve patient and provider experience, reduce health care costs and improve the health of the population to achieve a sustainable health care system [7]. To realize this, strategies to address medical workforce shortages and improve access to primary care in rural and remote Australia are urgently required.

The introduction of federally funded telehealth in Australian primary care settings during the coronavirus pandemic resulted in increased access to virtual care options for patients, and Australia’s Primary Health Care 10 Year Plan 2022-23 endorses further integration of telehealth into primary care settings to optimise service delivery [8]. This shift to virtual care presents an opportunity to explore telehealth-driven solutions to address rural health and workforce sustainability. Internationally, interventions which use offsite primary care providers to deliver ongoing care to patients via telehealth have been linked with improved health outcomes for patients and potential return on investment for rural practices [9, 10]. A US study of video telemedicine visits yielded a high degree of diagnostic concordance when compared with in-person visits for many clinical concerns [11]. Patients have also previously reported satisfaction with this type of service [10]. Furthermore, the implementation of virtual integrated care in areas facing workforce shortages may offer a means to moderate demands on the health system, by ideally managing patient needs in the context of primary care to prevent, defer, or reduce demands on secondary and tertiary sectors. For example, in a patient survey conducted as part of the Ontario Telemedicine Network Enhanced Access to Primary Care initiative evaluation, 4% of respondents reported that virtual care appointments replaced an emergency department visit [12]. However, there is limited evidence on virtual integrated care models to support rural and remote primary care practices, particularly in Australia [10]. There is thus a need to implement and evaluate this model of service delivery and to identify the service characteristics associated with safe, high-quality continuity of care. The aim of this pilot study was to implement and evaluate a Virtual Integrated Practice (VIP) program in the Australian primary care setting.

## Methods

### Study design

The pilot study was conducted in two western Queensland general practices, commencing in October 2021 with one rural practice and extending to an additional rural practice from November 2022. Evaluation included a retrospective review of service, billing and cost data, and an online survey for patients. Ethical approval was obtained from the University of Queensland Human Research Ethics Committee (Project number: 2021/HE002434).

The Queensland VIP Partnership Program was designed to address those issues most important to long-term practice sustainability and population health outcomes. As such, the aims and outcomes of the program are aligned with the Quadruple Aim of primary healthcare, which seeks to drive healthcare reform to improve (1) population health, (2) the patient experience of care, (3) reduce healthcare costs and (4) the work life of health providers [1], as well as core general practice features such as patient centricity and continuity of care.

### The Queensland Virtual Integrated Practice (VIP) model

In 2021, the Western Queensland Primary Health Network (WQPHN), Health Workforce Queensland (HWQ) and the UQ/MRI Centre for Health System Reform and Integration (CHSRI) partnered with three rural general practices to develop the Virtual Integrated Practice (VIP) Program. The model of service delivery involves an urban GP joining a rural general practice team to provide ongoing care to patients one or two days per week remotely via secure telehealth. The VIP GP joins the practice for a minimum of 12 months and works onsite for a short period (3–5 days) every 6-months. Participating practices offer telehealth appointments – preferably via videoconference – with the VIP GP to practice patients who predominantly attend their consultation from the practice. The VIP GP is provided with secure, remote access to the practice software/medical records to enable comprehensive, quality primary care. In addition, an avenue of communication is established between the VIP GP and practice staff e.g., Secure Messaging Service.

Development of the model utilised a co-creation approach between key stakeholders: WQPHN, HWQ, three Western Queensland GP practices and CHSRI, with a focus on accessible rural continuity of primary care as the shared goal. A rapid review of peer reviewed, and grey literature related to interventions which use offsite primary care providers to deliver care to patients in rural and remote communities remotely via telehealth was the first stage in the development of the model [10]. Secondly, a survey was emailed to each of the three general practices to determine (1) the clinical roles perceived as most needed in the practice; (2) the importance of receiving virtual support in these roles on a 5-point Likert scale (ranging from not important to critical) (3) support required for the practice-based workforce; (4) hours of virtual support required per fortnight; (5) on-the-ground infrastructure currently in place for virtual support and (6) patient- and practice-level facilitators and barriers foreseen to implementing virtual support in the practice. Finally, two workshops were conducted online with key stakeholders, namely the CHSRI and WQPHN, and general practice staff to discuss the survey results and develop the VIP model using co-creation methodology [13]. Principles of the partnership were collectively developed (Table 1).

**Table 1:** Principles of the Queensland Virtual Integrated Practice Partnership Program

Principles of Partnership:
1. A co-creation partnership
2. Quality training and service deliverables
3. Ongoing data collection related to implementation and outcomes of the program (patient/provider experience, service/administrative data, billing data/costs, VIP GP demographics)
4. A minimum 18-month VIP GP commitment, to ensure continuity of care is provided
5. Commitment to defray financial risk involved in participation for practices in the first 12 months
6. Involvement of all partners in the recruitment process for the VIP GPs, including Health Workforce Queensland, CHSRI and the general practice
7. The program doesn’t impact the existing rural GP workforce (i.e., VIP GP recruited from urban centres)
8. Willing to be involved in the scale up of the program in the respective primary health network area

### Virtual integrated practice program practices and GPs

Eligibility criteria for VIP general practices and GPs are presented in Tables 2 and 3. Practices were identified by the local primary health network based on need and suitability. More specifically, practices were selected if they were deemed vulnerable. Funding to support the business case and operations in the establishment phase of the program was provided to the VIP practice by the PHN. The GPs were recruited via local networks including The Royal Australian College of General Practitioners (RACGP) and existing rural locums. Interested GPs were invited to take part in a short interview with a member of the CHSRI project team to assess suitability and commitment. Following this, the potential VIP GP met virtually with the practice manager to further determine compatibility with the practice.

**Table 2:** Eligibility criteria for VIP general practices

Criteria:
1. Have high level digital literacy including video telehealth connectivity
2. Be an established practice with organisational and governance structures in place to support the pilot study
3. Have a practice team with an understanding of change management process, Medicare Benefits Schedule (MBS), and service delivery reform
4. Be committed to an 18-month proof-of-concept including regular feedback and data collection
5. Have room available for patients to attend appointments with VIP GP
6. Have nursing and administration support available as required.

**Table 3:** Eligibility criteria for VIP GPs

Criteria:
1. Possession of an urban Medicare provider number
2. Be vocationally Registered and have current registration with Australian Health Practitioner Regulation Agency
3. Be eligible for a Medicare provider number with the practice
4. Have experience with complex chronic disease management in the primary care setting
5. Have excellent working knowledge of Medicare Benefits Schedule (MBS)
6. Be able to work collaboratively as part of a multidisciplinary team, as well as autonomously
7. Have well-developed organisational and time management skills
8. Be skilled in the use of various telehealth technologies
9. Have excellent communication skills, in particular the ability to communicate safely and effectively via technology with persons from diverse backgrounds
10. Have excellent knowledge, understanding and sensitivity towards the social, economic, and cultural factors influencing health in rural and remote communities, including among Aboriginal and Torres Strait Islander peoples
11. Have worked at the practice for at least one week, or willing to travel to practice and deliver care on-site for one week prior to providing care virtually
12. Be able to commit to the Program for an extended period (≥ 18 months) to promote continuity of care.

Recruitment was confirmed by providing written consent. Following recruitment, practices and GPs attended an orientation session conducted by a member of the project team, which included training on Telehealth, the VIP Program, cultural awareness (where relevant) and data collection.

### Data collection

Service data was recorded by the VIP GP on an ongoing basis using a customised Microsoft Excel spreadsheet developed and provided by the research team (Additional Material 1). Service data included patient demographics (e.g., age), previous patient contact with the GP and the VIP service, reason for encounter, appointment location and practice staff support required.

Billing data was downloaded from the practice software quarterly by the practice manager and included the Medicare Benefits Schedule item number (which relates to the service that the Australian federal government subsidises), a description of service (i.e., phone or telehealth, length of service), payment type (i.e., private, Medicare, DVA, Health Care card or Workcover) and the amount charged. It is relevant to note that in Australia, GPs can bill for more than one service per visit (Additional Material 1).

Implementation cost data was recorded by the practice manager on an ongoing basis using a customised Microsoft Excel spreadsheet and included set-up costs (digital infrastructure), and recurrent costs (training, wages, accommodation, and travel costs for the onsite visits) (Additional Material 1).

Patients were invited to complete an anonymous survey about their experience via Qualtrics following each occasion of service (Additional Material 2). The survey was adapted from the Telemedicine Satisfaction Questionnaire, which is designed to evaluate the usefulness, satisfaction and quality of patient-clinician interaction over telemedicine technology [14]. Each practice was provided with a QR code and link to the survey to provide to patients and were later provided with a paper version of the survey in an attempt to increase participation. The survey included respondent characteristics (age, gender, if they had previously seen the VIP GP), consultation characteristics (type of consultation, place of attendance) and questions related to satisfaction and experience with the VIP service. Additional questions were added to the patient survey in October 2022 related to the perceived importance of care continuity, and emergency department avoidance. The survey included a free-text section where respondents could provide written feedback on their experience with the VIP GP and service.

Service, billing, and implementation cost data was requested from each practice quarterly by a member of the project team. De-identified responses to the patient survey were exported from Qualtrics on an ongoing basis.

### Data analysis

Service and billing data were used to produce patient characteristics (e.g., age, gender, and previous relationship with the VIP GP), the number of services billed under the VIP service and type of service provided (e.g., general GP consult, therapeutic procedure), percentage of appointments which required practice support and type of support required (e.g., nurse). Billing and cost data were used to calculate implementation (set-up) and recurrent costs. Responses to the patient experience survey were used to provide insight to acceptability and perceived quality of service from the perspective of end-users.

Data were analysed using the statistical software package IBM SPSS (Statistical Package for the Social Sciences) statistics (version 28.0.1, 2021) and are presented in results as mean ± standard deviation (SD), or number (percentage). Demographics of the survey responders were compared to all patients receiving a VIP consult using an unpaired t-test (age) and Fischer’s exact test (gender) (p < .05).

## Results

### Service, administration, and billing data

The VIP Partnership Program has been implemented in two rural general practices (Table 4). To date, there have been 1282 occasions of service provided, and more than half of these (53.0%) were repeat patient encounters. Patients were predominantly female (73.1%) and did not have their appointment at the practice (57.8%) (Table 5).

**Table 4:** VIP town and general practice characteristics

VIP	1	2
**Town**		
- Rurality^a^	Medium rural town	Very remote community
- MMM3-7^a^	MMM4	MMM7
- Population - town (n)	6,848	19,226
- Population - surrounding region (n)	12,688	22,613
- Primary care available in the town	3 general practices, hospital with ED	5 general practices, hospital with ED^b^
**Practice demographics**		
- Active patient population^c^ - n (%) female - % Aboriginal Torres Strait Islander - GP number - Other practice staff	5564 3106 (55.8) 227 (4.1) 5 Practice manager, reception staff, practice nurse	1715 771 (44.96) 106 (6.2) 1 Practice manager, reception staff, practice nurse
Program start date	October 2021	November 2022

^*a*^*Based on the Modified Monash Model* [15]

^*b*^*ED = emergency department* ^*c*^*Active patient population (seen 3 times in the last 2 years)*

**Table 5:** VIP patient characteristics (n = 1282 occasions of service)

Characteristic	Mean ± SD or n (%)
**Age (years)**	39.5 (20.4)
**Gender**	
Male	345 (26.9)
Female	937 (73.1)
**Aboriginal or Torres Strait Islander**	52 (4.1)
Location of VIP service*	
In clinic	540 (42.2)
Elsewhere	741 (57.8)
**Seen VIP GP > once**	551 (43.0)
**Used VIP service previously**	680 (53.0)

*Missing data n = 1.

Between October 2021 and December 2022, there were 1468 services provided, including general consults (n = 1197), therapeutic procedures (e.g., pregnancy test or ECG tracing) (n = 68), mental health treatment plans (n = 68), chronic disease management plans (n = 59), antenatal consults (n = 10) and script request (n = 1). The most frequently billed item number was a ‘longer consult, ≥ 6 mins telephone’ (item no. 91,891, n = 395), followed by a ‘short consult, ≤ 6 mins telephone’ (item no. 91,890, n = 357) and ‘attendance < 20 mins telehealth’ (item no. 91,800, n = 333). Among the 1282 occasions of service, less than 30% (n = 276) required support from other staff, most frequently a nurse, followed by administration and IT support. Few consultations required support from another GP in the practice (Table 6).

**Table 6:** Support staff required for the VIP service (n = 1282 occasions of service)

Support staff required^a^	n (%)
Nil support required	785 (74.2)
Nurse	151 (14.3)
Administration / IT	44 (4.2)
Practice manager	32 (3.0)
Care plan coordinator	23 (2.2)
Other staff member (e.g., dietitian)	19 (1.8)
Care plan coordinator and practice manager or nurse	4 (0.4)
Other GP	3 (0.3)

^a^Missing data n = 224

### Implementation cost data

The total cost of implementation (non-recurrent costs including IT and training) was $6911.50 and $2836.50 (Practice 1 and 2 respectively) (Additional Material 3). Recurrent costs included staff wages, GP onsite visits (travel and accommodation) and infrastructure (e.g., administration), the greatest of which were GP wages. The average cost per on-site visit to the practice (including travel and accommodation) was $1790.07 for Practice 1 and $1907.97 for practice 2.

### Patient experience survey

Survey respondents (n = 45, response rate 3.5%), mean age of 43.4 years (SD 17.97), and 80% female (n = 36), were statistically representative of the VIP patient population, and had predominantly attended the practice for a video consultation. All respondents indicated that the service met their needs and expectations very well, and that they were satisfied (9.3%) or highly satisfied (90.7%) with the care they received. Patients agreed (27.3%) or strongly agreed (72.3%) that they would use the service again (Table 7) and almost all (95.5%) respondents reported that the VIP service improved their access to the GP (Additional Material 4).

**Table 7:** Responses to the VIP patient experience survey (n = 45)

**Question**	n (%)
**What type of consultation did you have?**	
Video	42 (93.3)
Telephone	3 (6.7)
**Did you attend the consultation at your local general practice?**	
Yes	42 (93.3)
No	3 (6.7)
**Have you had a previous appointment with the GP you saw today?** ^**a**^	
Yes	21 (65.6)
No	11 (34.4)
**How well did your consultation today meet your needs and expectations?** ^**b**^	
Very well	24 (100.0)
Acceptable	0 (0.0)
Did not meet my needs and expectations	0 (0.0)
**Do you prefer virtual or face-to-face GP appointments?** ^**b**^	
Virtual	8 (33.3)
Face-to-face	13 (54.2)
No preference	3 (12.5)
**If you were unable to have this virtual GP appointment, would you have attended any of the following instead?** ^**c**^	
Emergency department	5 (21.7)
Other	18 (78.3)
**Please rate your satisfaction with the health and medical care received via video/telehealth today** ^**d**^	
Highly satisfactory	39 (90.7)
Satisfactory	4 (9.3)
Unsatisfactory	0 (0.0)

Missing data ^a^n=13; ^b^n=21; ^c^n=22; ^d^n=2

In the survey free text comment section, 40.0% of respondents left a comment (total of 18 comments received). Broadly, respondents described the previous lack of GP appointments available and reported improved access to the GP on account of the VIP service, with one patient noting that the virtual appointment prevented their attendance at the hospital to fill a script. One respondent indicated that they would prefer to see a doctor in-person.

*“So glad [VIP GP] worked. Not my usual GP but now have a script and didn’t have to wait at hospital for 4 hrs” – 50-59yo, F, telephone consultation*.

*“Amazing! So great to use for those who just can’t make it to the doctors in person on the day.” – 20-29yo, F, telephone consultation*.

*“I received great communication and understanding from the Doctor.” – 50-59yo, F, telephone consultation*.

*“If anything, Telehealth appointments with the [VIP] Doctor have made it so much easier… it takes away the daunting face to face contact” – 20-29yo, F, video consultation*.

*“Preferred appointment” – 40-49yo, F, video consultation*.

*“Very good way to have a consultation if you can’t get into your usual doctor. Not enough Drs in [VIP practice location]”– 50-59yo, F, video consultation*.

*“Such a lack of appointments face-to-face so great service to have.” – 60-69yo, F, video consultation*.

*“I like to talk and meet with an actually present doctor” – 70-79yo, F, video consultation*.

## Discussion

This pilot study demonstrates the feasibility and acceptability of a virtual integrated practice model of care within the Australian rural primary care setting, and its potential to support practice viability, improve patient access to care and reduce emergency department attendance. The Program leverages innovations in digital healthcare to improve workforce reach for rural general practices, building practice capacity and viability to better meet the health needs of communities, in line with the Primary Heath Care 10-Year Plan, National Medical Workforce Strategy and the Queensland Digital Strategy for Rural and Remote Healthcare [8, 16, 17]. Partnership, and a co-creation approach were key factors in the successful development and implementation of this pilot [18].

Access to health care is shaped by factors including acceptability, availability, affordability, and appropriateness, and is critical at both the patient and health system levels [19]. Implementation of the VIP Partnership Program in two Queensland general practices resulted in services spanning acute presentations and chronic disease management. This means that all patients have the potential to benefit from ongoing virtual primary care. Access to care, and an ongoing relationship with a primary care provider are crucial elements of continuity of care, which is linked with improved outcomes for patients, including higher patient satisfaction and lower hospitalisation rates [20]. Importantly, in this study, almost all survey respondents agreed that the VIP service improved their access to the GP, and that it was important or very important to see the same GP on an ongoing basis. In this model of care, the GP joins the practice for a minimum of 18 months, which naturally promotes care continuity, an enabler to structured care delivery in Australian rural primary care [21]. Improving care continuity in rural and remote communities is vital in reducing health inequalities between metropolitan counterparts [20]. This pilot study shows the potential for a virtual integrated care model to boost the capacity of Australia’s primary health care system to provide timely access to comprehensive care for Australians in rural and remote communities.

The VIP service was acceptable to patients in this study who elected to attend a virtual appointment. End users agreed that the service met their needs and expectations, and that they would use the service again. While some survey respondents indicated a preference for face-to-face appointments, almost all agreed that the service provided via telehealth was the same as for an in-person visit, and no respondents reported dissatisfaction with the service. Similar findings have been reported internationally [22]. In an evaluation of the Ontario Telemedicine Network Enhanced Access to Primary Care (EAPC) initiative, 98% of patients surveyed (n = 1705) felt that the virtual primary care visit was the same or better than in-person care, and 99.9% indicated that they would use virtual care again [12]. Similarly, in an evaluation of the Carrier Sekani Family Services primary care model, 64% of patients (n = 210) had used the service more than once, and 92% stated that they would recommend telehealth to others [23]. This is an important finding because the scalability and sustainability of a healthcare intervention are dependent in part on its acceptability [24]. Furthermore, patient acceptability and satisfaction are commonly used indicators for measuring quality in health care.

In recognition of the workforce crisis, the Stronger Rural Health Strategy outlines the need for “a sustainable, high quality health workforce that is distributed across the country according to community need, particularly in rural and remote communities”. The VIP Partnership Program is not designed to reduce or substitute face-to-face health services, but rather, to support the existing rural workforce. The Program marshals the urban GP workforce to flexibly extend workforce capacity without the risk of exacerbating the unequal distribution of general practice services [25]. Implementation of the service enhanced practice capacity to provide additional services, and therefore has the potential to reduce the workload of permanent GPs. This finding has been reported elsewhere; providers in a pharmacist-led telehealth intervention reported that the intervention did not create extra work, but rather, freed-up time for other activities. The benefit of a reduced workload for permanent GPs is two-fold; (1) burnout is the second most cited reason Australian GPs retire or leave general practice, and (2), a reduced workload creates more opportunity for permanent GPs to provide planned and structured care for people with chronic and complex conditions, which is typically more time consuming than other elements of care [26, 27].

While initial costs required to implement telehealth services have been shown to delay return on investment, models of service innovation which use offsite primary care providers to deliver longitudinal care via telehealth have ultimately been shown to be cost-effective for both patients and the health care system [28, 29]. At a health care system level, virtual integrated care models have the potential to reduce health care costs overall by ideally managing patients in primary care. Of interest, more than 20% of respondents in this study indicated that the virtual appointment replaced an emergency department visit, and similar findings have been reported internationally [12]. Furthermore, the VIP model promotes continuity of care, associated with reduced health care costs and acute care utilisation [30]. For patients, virtual care can reduce costs related to travel expenses and time off work, and patients in primary care have previously indicated a willingness to pay for virtual consultations [12, 31]. This study demonstrates the feasibility of the virtual integrated care model within the Australian primary care setting; with ongoing activity now focussed on a formal funding model for recurrent costs and scale-up.

Data from this pilot study has informed translation to an additional 20 rural general practices in three further Queensland Primary Health Networks in 2023. The ongoing evaluation framework includes semi-structured qualitative interviews with providers and other practice members, as well as identification of barriers and enablers for further state and national translation.

A strength of this pilot study is that it aimed to consider a range of perspectives, including the patient perspective. However, patients surveyed were only those who had attended a virtual appointment, not those who declined, which may have skewed findings related to the acceptability of virtual care. Additionally, the low response rate to the patient survey might be considered a limitation. This study was also limited to two general practices located in Queensland, Australia, and caution must be exercised against generalising these results.

## Conclusion

This pilot study demonstrates the feasibility of an innovative, digitally supported community-focussed, healthcare initiative to arrest the decline in rural general practice workforce, improve patient care access and support rural practice viability. It is acceptable to patients, fits well within the current Australian health care service delivery model, and maximises workforce flexibility and teamwork. Further work should investigate the optimal business model, full scope of clinical episodes of care, and barriers and enablers to broader national and international translation.

### Electronic supplementary material

Below is the link to the electronic supplementary material.

Supplementary Material 1

Supplementary Material 2

Supplementary Material 3

Supplementary Material 4

## Data Availability

The datasets used and/or analysed during the current study available from the corresponding author on reasonable request.
