# Supplementary material for: The Queensland Virtual Integrated Practice (VIP) partnership program pilot study: an Australian-first model of care to support rural general practice
Source: BMC Health Serv Res. 2023 Oct 31;23:1183. doi: 10.1186/s12913-023-10189-0 (PMC10617120; doi:10.1186/s12913-023-10189-0)
Supplement: Supplementary file 1 — Supplementary Material 1 [file 12913_2023_10189_MOESM1_ESM.docx]

**
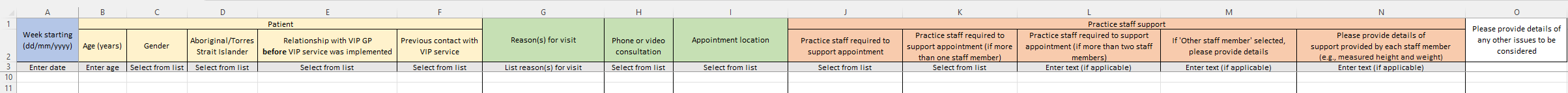
Additional Materials 1**

**Figure S1.** Service data spreadsheet template


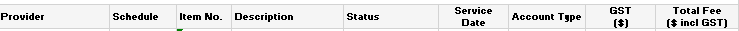


**Figure S2.** Service billing spreadsheet template

**
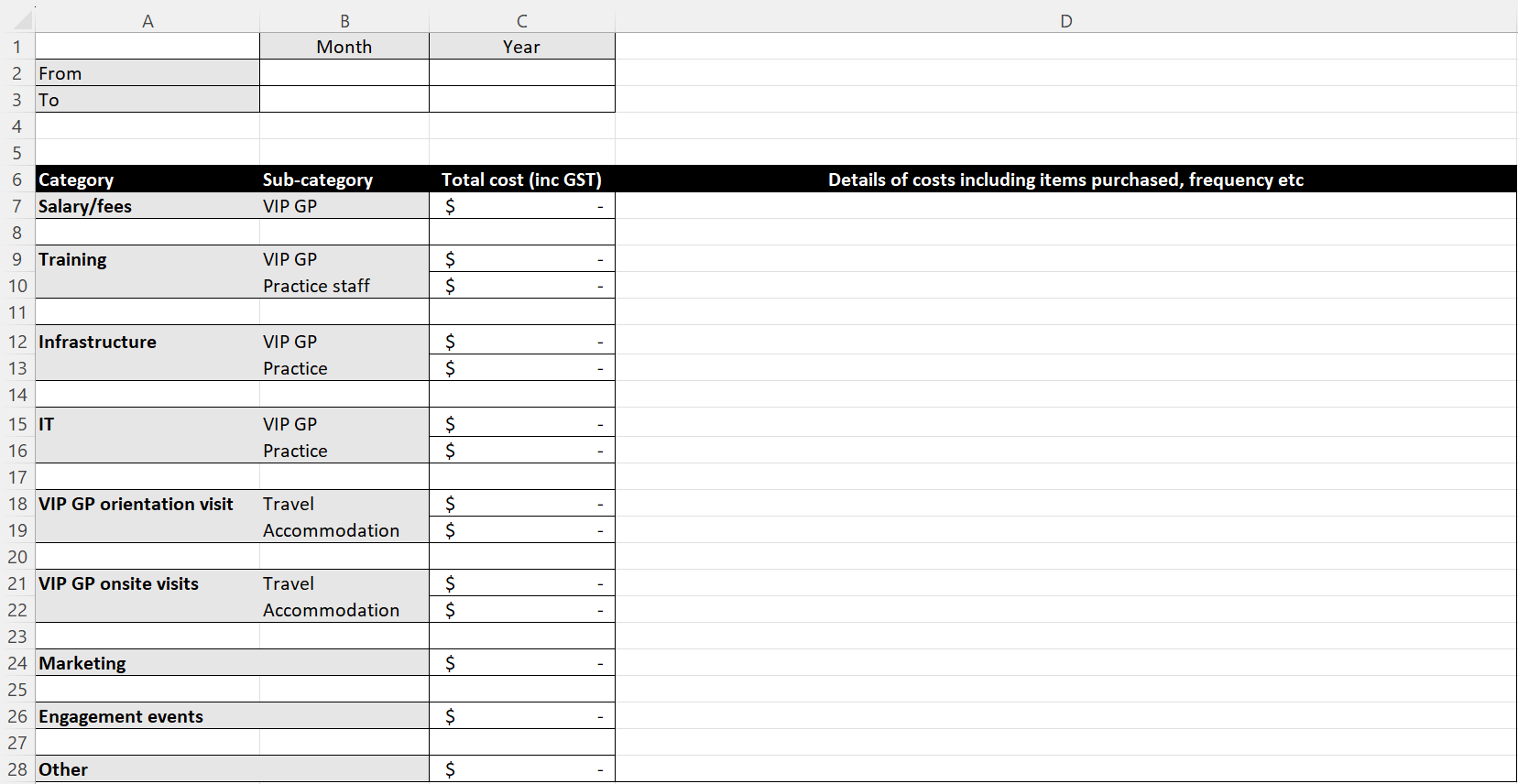
**

***Figure S3.*** *Cost data spreadsheet template*
