# Supplementary material for: The Queensland Virtual Integrated Practice (VIP) partnership program pilot study: an Australian-first model of care to support rural general practice
Source: BMC Health Serv Res. 2023 Oct 31;23:1183. doi: 10.1186/s12913-023-10189-0 (PMC10617120; doi:10.1186/s12913-023-10189-0)
Supplement: Supplementary file 2 — Supplementary Material 2 [file 12913_2023_10189_MOESM2_ESM.docx]

**VIP patient survey**

**1. What is your age (years):**

*Note. If you are completing this survey on behalf of someone else, please record the age of the person whom the appointment was for.*

**2. What is your gender:**

*Note. If you are completing this survey on behalf of someone else, please record the gender of the person whom the appointment was for.*

□ Male □ Female □ X (Indeterminate/Intersex/Unspecified)

**3. What type of consultation did you have with the GP today:**

□ Video □ Telephone

**4. Did you attend the consultation at your local general practice?**

□ Yes □ No

**5. Have you had a previous appointment with the GP you saw today?**

*Note. You may tick more than one answer for this question.*

□ In person □ Via video/telehealth □ Never

**6. How well did your consult today meet your needs and expectations?**

□ Very well □ Acceptable □ Did not meet my needs and expectations

**7. Do you prefer virtual GP appointments or face-to-face?**

□ Virtual □ Face-to-face □ No preference

**8. Rate the importance of seeing the same GP on an ongoing basis for your current condition (please tick one box below):**

| **Very important** | **Important** | **Moderately important** | **Somewhat important** | **Not important at all** |
| --- | --- | --- | --- | --- |
|  |  |  |  |  |

**9. If you were unable to have this virtual GP appointment, would you have attended any of the following instead?**

□ Emergency Department

□ Other (Please specify):

…………………………………………………………………………………………………………………….………………………….………………………………………………………………………………………………………………………………………………..…………………………………………………………………………………………………………

**10. Please rate your satisfaction with the health and medical care received via video/telehealth today**

□ Highly satisfactory □ Satisfactory □ Unsatisfactory

**11. For each statement please select an answer that best matches your experience of the video/telehealth appointment today (please tick one box for each statement below):**

|  | **Strongly agree** | **Agree** | **Neither agree or disagree** | **Disagree** | **Strongly disagree** |
| --- | --- | --- | --- | --- | --- |
| The video/telehealth appointment improved my access to the GP |  |  |  |  |  |
| I could easily talk to my GP and hear them clearly using  video/telehealth |  |  |  |  |  |
| I felt the service provided over the video/telehealth system was the  same as for an in-person visit |  |  |  |  |  |
| I felt comfortable communicating with the GP using the video/  telehealth system |  |  |  |  |  |
| I felt video/telehealth was an acceptable way to receive  healthcare services |  |  |  |  |  |
| I would use this GP video/ telehealth service again |  |  |  |  |  |

Please provide any further feedback you have to help improve this service:

…………………………………………………………………………………………………………………….………………………….………………………………………………………………………………………………………………………………………………..…………………………………………………………………………………………………………
