# Supplementary material for: The Queensland Virtual Integrated Practice (VIP) partnership program pilot study: an Australian-first model of care to support rural general practice
Source: BMC Health Serv Res. 2023 Oct 31;23:1183. doi: 10.1186/s12913-023-10189-0 (PMC10617120; doi:10.1186/s12913-023-10189-0)
Supplement: Supplementary file 3 — Supplementary Material 3 [file 12913_2023_10189_MOESM3_ESM.docx]

**Table S1. Implementation cost data**

| **Recurrent cost** | **Practice** | **Total ($AUD)** |
| --- | --- | --- |
| **GP wage** | Practice 1 | $1400/day |
|  | Practice 2 |  |
| **Staff and administration costs** | Practice 1 | $0.00 |
|  | Practice 2 | $3060 |
| **GP on-site visits** | Practice 1 | $12,530.46 (7 visits) |
|  | Practice 2 | $1907.97 (1 visit) |
| **Non-recurrent cost** | **Practice** | **Total ($AUD)** |
| **IT (e.g., laptop)** | Practice 1 | $3466.50 |
|  | Practice 2 | $2836.50 |
| **Staff training** | Practice 1 | $3445.00 |
|  | Practice 2 | $0.00 |
