# Supplementary material for: The Queensland Virtual Integrated Practice (VIP) partnership program pilot study: an Australian-first model of care to support rural general practice
Source: BMC Health Serv Res. 2023 Oct 31;23:1183. doi: 10.1186/s12913-023-10189-0 (PMC10617120; doi:10.1186/s12913-023-10189-0)
Supplement: Supplementary file 4 — Supplementary Material 4 [file 12913_2023_10189_MOESM4_ESM.docx]

**Table S2. Responses to VIP Patient Survey** **5-point Likert-scale questions**

| **Statement^a^** | **Very important** | **Important** | **Moderately important** | **Somewhat important** | **Not important at all** |
| --- | --- | --- | --- | --- | --- |
| Please rate the importance of seeing the same GP on an ongoing basis for your current condition | 15 (62.5) | 8 (33.3) | 1 (4.2) | 0 (0.0) | 0 (0.0) |
| **Statement^b^** | **Strongly agree** | **Agree** | **Neither agree nor disagree** | **Disagree** | **Strongly disagree** |
| The video/telehealth appointment improved my access to the GP | 34 (77.3) | 8 (18.2) | 2 (4.5) | 0 (0.0) | 0 (0.0) |
| I could easily talk to my GP and hear them clearly using video/telehealth | 39 (88.6) | 4 (9.1) | 1 (2.3) | 0 (0.0) | 0 (0.0) |
| I felt the service provided over the video /telehealth system was the same as for an in-person visit | 24 (54.5) | 19 (43.2) | 1 (2.3) | 0 (0.0) | 0 (0.0) |
| I felt comfortable communicating with the GP using the video/telehealth system | 32 (72.3) | 13 (29.5) | 0 (0.0) | 0 (0.0) | 0 (0.0) |
| I felt video/telehealth was an acceptable way to receive healthcare services | 28 (63.6) | 15 (34.1) | 1 (2.3) | 0 (0.0) | 0 (0.0) |
| I would use this GP video/telehealth service again | 32 (72.3) | 12 (27.3) | 0 (0.0) | 0 (0.0) | 0 (0.0) |

Missing data ^a^n=21; ^b^n=1
